# First description of *Echinococcus ortleppi* and cystic echinococcosis infection status in Chile

**DOI:** 10.1371/journal.pone.0197620

**Published:** 2018-05-17

**Authors:** Felipe Corrêa, Caroll Stoore, Pamina Horlacher, Mauricio Jiménez, Christian Hidalgo, Cristian A. Alvarez Rojas, Guilherme Figueiredo Barros, Henrique Bunselmeyer Ferreira, Marcela Hernández, Gonzalo Cabrera, Rodolfo Paredes

**Affiliations:** 1 Escuela de Medicina Veterinaria, Facultad de Ecología y Recursos Naturales, Universidad Andres Bello, Santiago, Chile; 2 Centre for Animal Biotechnology, Faculty of Veterinary and Agricultural Sciences, The University of Melbourne, Parkville, Australia; 3 Institute of Parasitology, University of Zurich, Zurich, Switzerland; 4 Laboratório de Genômica Estrutural e Funcional, and Laboratório de Biologia Molecular de Cestódeos, Centro de Biotecnologia, UFRGS, Porto Alegre, RS, Brazil; 5 Laboratorio de Biología Periodontal, Facultad de Odontología Universidad de Chile and Faculty of Health Sciences, Universidad Autónoma de Chile, Santiago, Chile; 6 Programa de Biología Celular y Molecular, Instituto de Ciencias Biomédicas, Facultad de Medicina, Universidad de Chile, Santiago, Chile; University of Oxford, UNITED KINGDOM

## Abstract

Cystic echinococcosis (CE), a parasitic disease caused by the cestode *Echinococcus granulosus sensu lato (s*.*l*.*)*, is a worldwide zoonotic infection. Although endemic in Chile, information on the molecular characteristics of CE in livestock remains scarce. Therefore we aimed to describe the status of infection with *E*. *granulosus s*.*l*. in cattle from central Chile and also to contribute to the study of the molecular epidemiology of this parasite. According to our results, the prevalence of CE is 18.84% in cattle, similar to previous reports from Chile, suggesting that the prevalence in Santiago Metropolitan area has not changed in the last 30 years. Most of the cysts were found only in lungs (51%), followed by concurrent infection in liver and lungs (30%), and only liver (19%). Molecular characterization of the genetic diversity and population structure of *E*. *granulosus s*.*l*. from cattle in central Chile was performed using a section of the cytochrome c oxidase subunit 1 (*cox1*) mitochondrial gene. *E*. *granulosus sensu stricto (s*.*s*.*)* (G1-G3 genotypes) was confirmed by RFLP-PCR to be the dominant species affecting cattle (284 samples/290 samples); we also report for the first time in Chile the presence of *E*. *ortleppi* (G5 genotype) (2 samples/61 samples). The Chilean *E*. *granulosus s*.*s*. parsimony network displayed 1 main haplotype. Additional studies using isolates from many locations across Chile and different intermediate hosts will provide more data on the molecular structure of *E*. *granulosus s*.*s*. within this region. Likewise, investigations of the importance of *E*. *ortleppi* in human infection in Chile deserve future attention.

## Introduction

Genotyping of human and livestock CE is useful to assess the information on parasite transmission patterns for epidemiological purposes and detection of species or genotypes that can infect humans. *Echinococcus granulosus sensu lato (s*.*l*.*)* is composed of: *E*. *granulosus sensu stricto* (*s*.*s*.; G1/G2/G3 genotypes), *E*. *equinus* (G4 genotype), *E*. *ortleppi* (G5 genotype), *E*. *canadensis* (G6/G7/G8/G10 genotypes) and *E*. *felidis* (‘lion strain’) [1]. Moreover a high variability has been discovered within species of the *E*. *granulosus s*.*l*. complex when sequencing longer fragments of DNA. For example based on full length of the cox and/or nad genes multiple haplotypes exist within *E*. *granulosus s*.*s* [2], *E*. *ortleppi* [3] and *E*. *canadensis* G6/7 [4]. Molecular data for the investigation of genotypes of *E*. *granulosus s*.*l*. in Chile is limited; while *E*. *granulosus s*.*s*. has been reported in humans and cattle [5–8], *E*. *canadensis* (G6) was reported in 1 human sample [5]; other genotypes or species have never been described so far. While *E*. *granulosus s*.*s*. is also the most common genotype found in Chilean neighbor countries, *E*. *ortleppi* is the second most common and has been reported in Argentina, Uruguay and Brazil [1] suggesting that it could also be present in Chile.

Cystic echinococosis (CE), also known as hydatid disease, is a zoonotic disease with worldwide distribution [9–12]. It is caused by the larval stage of *E*. *granulosus s*.*l*., a cestode parasite able to infect a variety of intermediate hosts, including livestock, such as sheep, goats, cattle, camels, buffaloes and pigs, and also humans [13]. The hydatid cysts of *E*. *granulosus s*.*l*. develop as unilocular fluid-filled bladders within the infected internal organs (mainly liver and lungs) of the intermediate hosts.

In South America, CE is endemic in the Andean and South regions, including South of Brazil, Argentina, Chile, Uruguay and Peru [12, 13]. The epidemiological status in South America is not fully understood and broad data is scarce, especially in Uruguay and Chile [1]. In Chile, according to national surveillance data, the surgical incidence has remained steady at around 2/100,000 inhabitants since the early 1990s. However, based on hospital discharge records of patients who underwent surgical treatment, the incidence was 4.68-5/100,000 inhabitants in the period 2009–2014, this difference supports several authors who have suggested the existence of sub notification of this disease in Chile [14–16]. In human patients in Chile, CE is responsible for direct monetary costs estimated in US$ 7,379 per patient in a private clinic, which involves diagnosis, hospitalization and treatment[16].

In cattle, the annual number of CE confirmed cases remains stable ranging between 18–20% of total cattle slaughtered within the last 2 years, ranked as the second cause of offal condemnation in this species after the infection with *Fasciola hepatica* (fascioliasis)[17]. In livestock the largest economic impacts of CE have been suggested to be productivity losses and costs for condemnation of viscera at meat inspection [10, 18–21]. By the year of 2011, in Chile it was estimated a loss of US$ 1,449,931 only caused by liver condemnation in cattle [16].

In Chile, a number of high risk behaviors/factors that favors the transmission of the parasite remain present. Deficient responsibility in dog ownership [16], absence of treatment of dogs against helminths, permanence of habit of feeding dogs with uncooked offal, high rates of echinococcosis in dogs and more importantly, home slaughter [22] are considerable elements that favor the risk of exposure to CE.

Despite its great economic and public health significance, CE receives little attention in Chile [23, 24]. Thus, the objective of the present study was to characterize the prevalence, organ distribution and fertility of hydatids in cattle, and to enlarge our knowledge on the molecular identity of *E*. *granulosus s*.*l*. infecting this important livestock species slaughtered in Santiago, Chile.

## Materials and methods

### Study design

A descriptive study was conducted to determine the prevalence, organ distribution and characteristics of hydatid cysts in cattle at a slaughterhouse in Santiago, Chile. The Universidad Andres Bello Bioethics Board approved the study protocol (protocol number 016/2016). For this reason, one slaughtering-day visit per week was scheduled from July 2013 to June 2015. It is important to take into account that animals slaughtered in Santiago could have been originated from the same region or its southern area. The internal organs (lungs, liver, heart, spleen and kidney) of slaughtered animals were carefully examined by official veterinarian inspectors for the presence CE (and other pathologies). Age and sex of each animal was recorded; two age-based groups were distinguished: Group 1 (≤4 years old) and Group 2 (>4 years old). Cysts found were removed from each infected organ and transported in iced boxes within 2 hours to the laboratory for further examination. To evaluate cyst fertility, cysts were inspected for the presence of protoscoleces [25]. Protoscoleces and/or germinal layer from both fertile and infertile cysts were washed three times with sterile saline solution, fixed in 70% ethanol and stored at 4°C until genomic DNA isolation.

### Data analysis

Data was entered into a Microsoft Excel 2010 database, followed by analysis using SPSS V.15. The Chi-squared test was used to compare the prevalence of hydatid cysts among cattle of different age and sex. Confidence intervals were calculated using Graphpad Prism 6. A statistically significant association between variables is considered to exist if the p value is <0.05.

### Molecular and sequence analysis

Protoscoleces and/or germinal layers from each individual cyst were washed five times in sterile-distilled water by centrifugation to remove the ethanol, and genomic DNA (gDNA) was extracted using a DNA Purification System Kit (Promega, Madison, WI, USA). Concentration of each DNA sample was measured using Take3 micro-volume plate (Biotek Synergy™, Winooski, VT, USA), and they were stored at -20°C until PCR amplification. A 444-bp fragment of the cytochrome c oxidase subunit 1 (*cox1*) was amplified using a gene specific pair of primers (5’-TTTTTTGGGCATCCTGAGGTTTAT-3’) and (5’-TAAAGAAAGAACATAATGAAAATG-3’) [26]. Amplification was carried out in a 25 μl final volume containing 50–100 ng of gDNA, 200 μM of dNTP mix, 3 mM of MgCl_2_, 10 pmol of each primer, and 1.5 U of Taq DNA polymerase. PCR amplification of isolated DNA samples plus positive and negative controls was performed in a thermocycler (Mastercycler, Eppendorf, Germany) under the following cycling conditions: initial denaturation cycle at 95°C for 5 min, followed by 35 cycles of 55 s at 94°C, 55 s at 54°C and 60 s at 72°C with a final step at 72°C for 10 min. The PCR products were later analyzed by electrophoresis on 1.2% agarose gels, stained with SYBR safe and visualized under UV light (G:BOX, Syngene). In order to determine if the samples were only *E*.*granulosus s*.*s*., the positive samples to PCR were then subjected to PCR-restriction fragment length polymorphism analysis (PCR-RFLP) with 10 U of the *Alu*I restriction endonuclease for 4 h, according to the manufacturer's instructions (Fermentas, Germany), in a final volume of 20 μl. The restriction fragments were fractionated in 1.2% agarose gels, stained with SYBR safe and visualized under a UV light (G:BOX, Syngene). PCR-RFLP patterns were compared with reference patterns for *E*. *granulosus s*.*s* [27].

The PCR products of *cox1* were purified and subjected to sequencing with both primers used for amplification. The acquired sequences were manually inspected and aligned using Geneious (version 8.0.4) (http://www.geneious.com, [28]) with reference sequences for each genotype: G1 and G3 complete mitochondrial *E*. *granulosus s*.*s*. genome (AB786664, KJ559023), G2 mitochondrial *E*. *granulosus s*.*s*. gene sequences (AJ237633, M84662), G5 mitochondrial *E*.*ortleppi* gene sequence (M84665) and G6 and G7 complete mitochondrial *E*. *canadensis* genome (AB208063, AB235847). A network of *cox1* mitochondrial haplotype using statistical parsimony was drawn by TCS 1.2 software [29] for an schematic representation of the diversity and relationships among the different haplotypes found.

## Results

### Prevalence

From 2,961 cattle examined, the overall prevalence of CE was 18.84% (CI95% 17.45–20.30) (Table 1). Cyst prevalence was significantly higher in cattle over 4 years old compared to those under 4 years of age (χ2 = 141.6, p<0.0001), and also between the same age ranges in males (χ2 = 18.36, p< 0.0001) and females (χ2 = 94.83, p<0.0001). Even though there is a significant difference in the prevalence regarding sex (χ2 = 29.45, p<0.0001), no difference was found between male and female cattle of the same age ranges (Females: χ2 = 0.0066, p = 0.9355; Males: χ2 = 0.01381; p = 0.9065).

**Table 1:** CE prevalence by sex and age, in cattle slaughtered Santiago, Chile.

		Examined	Infected	Prevalence % [CI_95%_]	*χ*^2^	*p*
Overall		2961	558	18.84 [17.45–20.30]		
Age range						
<4 years		2213	307	13.87 [12.46–15.38]	141.6	<0.0001*
>4 years		748	251	33.56 [30.18–37.07]		
Sex						
Female		471	131	27.81 [23.81–32.1]	29.45	< 0.0001*
Male		2490	427	17.15 [15.69–18.69]		
Sex in each age range						
<4 years:	Female	132	18	13.64 [8.29–20.69]^a^	0.0066	0.9355
	Male	2081	289	13.89 [12.43–15.49]^b^		
>4 years:	Female	339	113	33.33 [28.33–38.5]^a^	0.01381	0.9065
	Male	409	138	33.74 [29.19–38.5]^b^		

Results expressed as absolute or relative (%) frequencies.

CI: Confidence interval

*: statistically significant difference (p<0,05)

a: Significant difference between females of different age range (χ2 = 18.36; p< 0.0001)

b: Significant difference between males of different age range (χ2 = 94.83; p<0.0001)

### Organ distribution and cysts characterization

From the total 558 cattle harboring one or more hydatid cysts in their internal organs, 284 had cysts only in their lungs (51%), 108 only in their livers (19%), while the rest of the 168 infected cattle (30%) had concurrent infection in both organs. The analysis of fertility revealed that 430 cysts were infertile (79%) and 29 fertile (6%). For 101 (15%) cysts the fertility test was not performed due to their small size (diameters smaller than 1.5 cm). The highest proportion of fertile cysts occurred in the lungs of infected cattle.

### Molecular cyst characterization

Partial PCR amplification of *cox1* yielded an expected 444-bp fragment for 290 of all the DNA samples of hydatid cysts characterized by PCR-RFLP, which resulted in 98% (284/290) identified as *E*. *granulosus s*.*s*. (G1/G2/G3 genotypes), while 2%(6/290) of the samples were identified as G4-G10 genotypes (Table 2).

**Table 2:** *Echinococcus* species in cattle hydatid cysts detected in the present study based on restriction length polymorphism-PCR analysis.

Species identified by molecular analysis	No. isolates	Organ	
		Liver	Lungs
*E*. *granulosus s*.*s*. (G1,G2,G3)	284	86	198
Other *E*. *granulosus s*.*l*. species (G4-G10)	6	2	4
Total	290		

To confirm the identified genotypes, 61 samples were sequenced for *cox1* mtDNA. From the *cox1* nucleotide sequences obtained from DNA isolates, a 345-nucleotide consensus sequence was used for comparative analysis. Alignments of the sequences determined herein with those of known genotypes of *E*. *granulosus* revealed the existence of 59 *E*. *granulosus s*.*s*. (G1-G3) and 2 *E*. *ortleppi* (G5) samples out of 61 in the studied area.

In total, 11 different haplotypes were detected among the 59 *E*. *granulosus s*.*s*. isolates and were deposited in GenBank under the accession numbers MF421702-MF421712, while an unique haplotype was identified among the 2 *E*. *ortleppi* isolates, and it was deposited in GenBank under the accession number MF421713 (S1 Fig).

## Discussion

The 18.84% prevalence for CE in cattle in the present study is similar to previous reports from Chile [30–32]. Interestingly, the report by Luengo and Olivares [30] was based on samples obtained from the same area included in our study, suggesting that the prevalence in Santiago Metropolitan area has not changed in the last 30 years.

A higher prevalence of CE in old cattle (>4 years) compared to young ones (<4 years) was observed in the present study, which is similar to other reports [33–35]. Several studies have suggested that this difference can be explained by the prolonged exposure of older animals to a larger number of infective stages of the parasite, favoring the development of cysts [36–38].

In our study, the largest proportion of hydatid cysts occurred in the lungs rather than other organs, which is in agreement with previous publications in different countries: 63.7% [37] and 57.8%[39] in Iran, 55.2% [40] and 71.6% [38] in Ethiopia, 93.8% in Argentina and 81.2% in Spain [41], and 77.7% in Brazil [42]. But in disagreement with other investigations in where the liver is the most common location for the cyst; 61.8% in Sudan [43] and 62.58% in Tunisia [33]. The cattle slaughtered age and the hydatids strains variations could be attributable as the cause for this difference. Also, it has been suggested that liver capillaries have widened in older animals, which allows most oncospheres to pass from the liver to the lungs [44].

Our overall finding of 79% of hydatid cysts were infertile are comparable to results obtained by Negash *et al*. in which 71.5% were infertile [38], Pednekar *et al*. with 81.25% [45], and Balbinotti *et al*. with 91.2% of infertile cysts [42]. This low fertility frequency supports previous arguments proposed by several researchers that consider sheep to have a greater role as an intermediate host of cystic echinococcosis rather than cattle [46, 47]. Interestingly, a study conducted in Sudan demonstrated a higher (77%) fertility rate of hydatid cyst in cattle, corresponding mostly to G6 and G5 strain [43]. However, the findings in the present study of a high overall prevalence (in 18.84% of cattle) and fertility of 6%, is in line with previous reports (16.4 infected/14.2% fertility) [18, 37, 48], implying that cattle are still important as a source of infection to the definitive hosts of this parasite.

In the present study, lungs were the most common organ for the development of fertile hydatids, which is in agreement with previous reports (14% fertility in lungs)[48, 49]. Its been suggested that the softer consistency of lung tissue compared with other organs possibly favors the development and fertility of cysts [48, 49]. Nevertheless, this finding is in contrast with other report [20]; which can be explained by the presence of different *E*.*granulosus s*.*l* strains in each location [18, 39, 44, 50].

To date, only *E*. *granulosus* s.s. has been reported in intermediate hosts in Chile: G1 in cattle [6] and humans [5]; G3 in cattle [6]; a number of haplotypes of *E*. *granulosus* s.s. [8] and *E*. *canadensis* G6 in a single human isolate [5]. In the present study, the G1-G3 cluster and microvariants of this group was predominant (61/63) among all the isolates studied. This is in agreement with the broad distribution of this species since the G1-G3 complex is the most frequent genotype identified in livestock [51–53] and humans [54]. Although in some countries of North Africa such as Sudan and Mauritania, G6 is the most common genotype in sheep, cattle, camels and human [43, 55]. In the present study, genetic characterization of *E*. *granulosus* isolates employing mitochondrial *cox1* sequences revealed that the *E*. *ortleppi* is also present in cattle in Chile. To our knowledge, this is the first report of *E*. *ortleppi* infection in any intermediate host in Chile, which sets the basis to further explore whether *E*. *ortleppi* is also responsible for human infection as reported in other countries.

The structure of the parsimony network generated using the 59 *cox1 E*. *granulosus* G1 isolates consisted of 11 haplotypes (MF421702-MF421713), MF421707 being the most dominant with 43 isolates. A BLAST search showed that the sequence of the haplotype MF421707, had a 100% identity with those of the globally distributed *E*. *granulosus* s.s haplotype described from Egypt [56], United Kingdon [57], Tunisia [58], Mongolia [59], Russia [60].

In conclusion, the findings reported here show that CE is widespread in cattle in Santiago and its Southern area. Our results shows that cattle may have an important role in the life cycle of this zoonotic disease and highlight the presence of potential hazards of transmission to the human population and other intermediate hosts of the studied area. More importantly we detect the presence of *E*. *ortleppi* in Chile for the first time, further epidemiological studies on the abundance of this species and its role in human infection in Chile is needed.

## Supporting information

S1 Fig Haplotypes network of *cox1* mtDNA (345bp). The network depicts 11 haplotypes of *E*. *granulosus s*.*s*. corresponding to 59 of the isolates that were analyzed, and 1 haplotype of *E*. *ortleppi*. The size of each figure is proportional to the frequency to the respective haplotype found. Each mutation event is represented through on the lines by a dash.
(TIFF)
