# Supplementary figures and images for: First description of Echinococcus ortleppi and cystic echinococcosis infection status in Chile
Source: PLoS One. 2018 May 17;13(5):e0197620. doi: 10.1371/journal.pone.0197620 (PMC5957416; doi:10.1371/journal.pone.0197620)

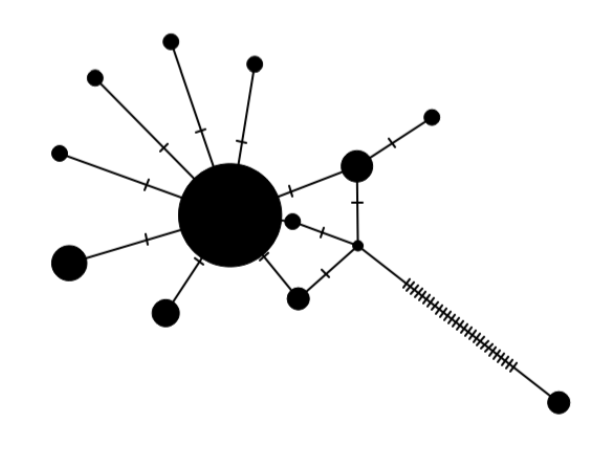

Supplement: S1 Fig — The network depicts 11 haplotypes of E. granulosus s.s. corresponding to 59 of the isolates that were analyzed, and 1 haplotype of E. ortleppi. The size of each figure is proportional to the frequency to the respective haplotype found. Each mutation event is represented through on the lines by a dash. (TIFF) [file pone.0197620.s001.tiff]
